# Deciphering the evolving niche interactome of human hematopoietic stem cells from ontogeny to aging

**DOI:** 10.3389/fmolb.2024.1479605

**Published:** 2024-12-04

**Authors:** Cong Feng, Haoyan Fan, Ruxiu Tie, Saige Xin, Ming Chen

**Affiliations:** ^1^ Department of Bioinformatics, College of Life Sciences, Zhejiang University, Hangzhou, China; ^2^ Bioinformatics Center, The First Affiliated Hospital, Zhejiang University School of Medicine, Hangzhou, China; ^3^ Zhejiang University-University of Edinburgh Institute, Zhejiang University, Haining, China; ^4^ Bone Marrow Transplantation Center, the First Affiliated Hospital, Zhejiang University School of Medicine, Hangzhou, China; ^5^ Department of Hematology-Oncology, Taizhou Hospital of Zhejiang Province, Linhai, China

**Keywords:** hematopoietic stem cell, hematopoietic niche, single-cell RNA sequencing, cell-cell communication, aging

## Abstract

Hematopoietic stem cells (HSC) reside within specialized microenvironments that undergo dynamic changes throughout development and aging to support HSC function. However, the evolving cell-cell communication networks within these niches remain largely unexplored. This study integrates single-cell RNA sequencing datasets to systematically characterize the HSC niche interactome from ontogeny to aging. We reconstructed single-cell atlases of HSC niches at different developmental stages, revealing stage-specific cellular compositions and interactions targeting HSC. During HSC maturation, our analysis identified distinct patterns of ligand-receptor interactions and signaling pathways that govern HSC emergence, expansion, and maintenance. HSC aging was accompanied by a decrease in supportive niche interactions, followed by an adaptive increase in interaction strength in old adult bone marrow. This complex aging process involved the emergence of interactions associated with inflammation, altered stem cell function, and a decline in the efficacy of key signaling pathways. Our findings provide a comprehensive understanding of the dynamic remodeling of the HSC niche interactome throughout life, paving the way for targeted interventions to maintain HSC function and promote healthy aging. This study offers valuable insights into the intricate cell-cell communication networks that govern HSC behavior and fate, with implications for hematological disorders and regenerative medicine.

## 1 Introduction

Cell-cell communication between stem cells and their microenvironment is a crucial driving force for various developmental processes, including cell growth, differentiation, homeostasis, and aging (Armingol et al., 2021). The hematopoietic niche, which provides a supportive microenvironment for HSC, undergoes multiple tissue/organ switches during the ontogeny, expansion, maturation, and aging of HSC (Orkin and Zon, 2008). These switches are accompanied by dynamic changes in the cellular composition and molecular landscape of the niche, which play a critical role in regulating HSC function and fate. After colonizing the bone marrow, HSC continue to interact with niche cells to maintain their self-renewal and multi-lineage differentiation capacities until aging (Pinho and Frenette, 2019; Hofmann and Kokkaliaris, 2024). However, the dynamic changes in the niche interactome throughout HSC lifespan and the potential regulatory mechanisms remain largely unexplored, highlighting the need for a comprehensive understanding of the evolving hematopoietic niche.

In the human embryo, HSC originate from the aorta-gonad-mesonephros (AGM) region through endothelial-to-hematopoietic transition (EHT) at Carnegie stages 13–17 (4–6 weeks) (Lancrin et al., 2009; Bertrand et al., 2010; Boisset et al., 2010). Recently, single-cell RNA sequencing (scRNA-seq) has provided unprecedented insights into the dynamic cellular development and complex interactions during developmental hematopoiesis. Using single cell techniques, the human AGM niche was revealed to comprise endothelium, mesenchymal stem/stromal cells (MSC), epithelium, and intra-aortic hematopoietic clusters (IAHCs) (Zeng et al., 2019; Calvanese et al., 2022). Ligand-receptor expression analysis has revealed intricate interactions between sub-aortic mesenchymal populations and hemogenic endothelial cells (HECs) through key signaling pathways such as DLK1-NOTCH1, SPP1-CD44, and WNT2B-FZD4 (Zeng et al., 2019). The NOTCH signaling pathway plays a critical role in HSC ontogeny, with NOTCH1 and NOTCH2 receptors expressed on HECs and their ligands (DLL4, JAG1) expressed on neighboring endothelial and stromal cells (Gama-Norton et al., 2015; Souilhol et al., 2016). In mice, urogenital ridges (UGRs) express several integrins, insulin growth factors, Kit, and Tgfβ signaling components that influence hematopoietic development (Lummertz da Rocha et al., 2022). Additionally, somites, endothelium, and macrophages have been shown to provide essential factors (such as SCF, BMP4, and IL-3) for HSC development (Nguyen et al., 2014; Mariani et al., 2019; Wattrus et al., 2022), emphasizing the complex and multifaceted nature of the AGM niche in supporting HSC.

After transdifferentiation and fate determination in the AGM region, nascent HSC migrate through the blood circulation to the fetal liver (FL) for expansion. The FL hematopoietic microenvironment is more complex, including endothelium, stromal cells, hepatocytes, and immune cells (Lewis et al., 2021). Cytokines and growth factors like SCF, TPO, ANGPTL2/3, and IGF2 secreted by endothelium and hepatocytes are required for FL hematopoietic stem/progenitor cell (HSPC) expansion (Zhang et al., 2006; Chou and Lodish, 2010; Sugiyama et al., 2011). These factors activate signaling pathways, such as PI3K/AKT and JAK/STAT, in HSPCs to promote their proliferation and maturation. A recent spatial transcriptome study also indicated that arterial and sinusoidal endothelial cells in FL produce Notch and Cxcl12 signals to support HSC (Lu et al., 2021). The WNT/β-catenin signaling pathway is essential for HSC expansion in the FL, with WNT ligands secreted by stromal cells and hepatocytes (Luis et al., 2009; Ruiz-Herguido et al., 2012).

Following expansion in the FL, HSC migrate to the fetal bone marrow (FBM), where a diverse array of niche cells, including MSC, endothelial cells, osteolineage cells (OLCs), and adipocytes, are detected (Jardine et al., 2021; Hofmann and Kokkaliaris, 2024). Systematic cell-cell communication analysis suggested that there are more interactions between endothelial and reticular cells of FBM and HSC to provide NOTCH signaling ligands (JAG1, JAG2, DLL4 and DLK1, etc.) required for HSC development (Zheng et al., 2022), highlighting the evolving nature of the hematopoietic niche and its adaptation to support HSC function in different developmental stages. The TGF-β signaling pathway also plays a crucial role in HSC maturation, with TGF-β1 secreted by FBM stromal cells regulating HSC quiescence and differentiation (Blank and Karlsson, 2015). TGF-β1 binds to its receptors on HSCs, activating SMAD signaling and inducing cell cycle arrest and differentiation.

As HSC transition from fetal to adult, they acquire a more quiescent phenotype and expressing key transcription factors, such as SOX17 and CEBPα (Kim et al., 2007; Ye et al., 2013). This shift is accompanied by significant changes in the bone marrow niche, which adapts to support the long-term maintenance and function of adult HSC. The CXCL12-CXCR4 signaling axis is critical for HSC maintenance, with CXCL12 secreted by MSCs, endothelial cells, and OLCs (Sugiyama et al., 2006; Ding and Morrison, 2013). CXCL12 binds to CXCR4 on HSCs, activating downstream signaling pathways, such as PI3K/AKT and MAPK, to promote HSC survival and retention in the niche. The SCF-KIT signaling pathway also plays a vital role in HSC maintenance, with SCF expressed by perivascular and endothelial cells (Ding et al., 2012). SCF binding to KIT on HSC activates signaling cascades, such as PI3K/AKT and MAPK, to support HSC survival and self-renewal. Additionally, the Hippo-YAP signaling pathway regulates HSC maintenance, with YAP activity in stromal cells promoting HSC proliferation and regeneration (Jansson and Larsson, 2012). Niche cells with active YAP secrete factors that stimulate HSC proliferation and regeneration, such as CXCL12 and SCF.

During aging, the HSC niche undergoes significant remodeling, contributing to HSC functional decline and differentiation skewing (Matteini et al., 2021). For example, decreased vascular density and organization lead to reduced availability of HSC-supportive factors (SCF, CXCL12) (Itkin et al., 2016; Poulos et al., 2017), while decreased MSC and shifted differentiation towards adipogenesis rather than osteogenesis also impact HSC function (Naveiras et al., 2009). These changes in niche cell composition and function lead to altered signaling in aging HSCs, such as reduced CXCL12-CXCR4 and SCF-KIT signaling, contributing to their functional decline. Moreover, increased levels of pro-inflammatory cytokines (interleukin, TNF-α) during aging can promote HSC proliferation and differentiation, depleting the HSC pool (Kovtonyuk et al., 2016; Pietras et al., 2016). These inflammatory cytokines activate signaling pathways, such as NF-κB and MAPK, in HSC, leading to their proliferation and differentiation at the expense of self-renewal. Furthermore, the accumulation of DNA damage and epigenetic alterations in aging HSC, which can be influenced by niche-derived factors, contributes to their functional decline (Wendorff et al., 2022; Kasbekar et al., 2023). These age-related changes in the HSC niche contribute to impaired hematopoiesis and increased susceptibility to hematological disorders, emphasizing the critical role of the niche in maintaining HSC function throughout lifespan.

Young and aged HSCs exhibit distinct functional and molecular characteristics. Young HSCs have a higher proliferation potential and a balanced differentiation capacity towards both myeloid and lymphoid lineages, whereas aged HSCs show reduced proliferation and a skewed differentiation towards the myeloid lineage (de Haan and Lazare, 2018). Depletion of myeloid-biased HSC can rejuvenate aged immunity (Ross et al., 2024). Aged HSCs also display altered retention and mobilization properties, with increased mobilization from the bone marrow niche and reduced homing ability (Xing et al., 2006). This is associated with changes in the expression of adhesion molecules, such as integrins and selectins, on aged HSCs and their niche cells (Matteini et al., 2021). Furthermore, aged HSCs exhibit distinct marker expression profiles, with increased expression of CD150 and reduced expression of ATF4 and CD49f (Sun et al., 2021; Hammond et al., 2023). Aged HSC exhibit a decrease in the frequency of polar cells, leading to a preferential shift towards symmetric self-renewing divisions. This change in cell polarity and division mode is controlled by the activity of the small RhoGTPase Cdc42, with aged HSC undergoing more symmetric divisions that result in daughter stem cells with reduced regenerative capacity and lymphoid potential (Florian et al., 2018). In contrast, young polar HSC undergo primarily asymmetric divisions. The asymmetric sorting of Cdc42 during cell division plays a mechanistic role in determining the potential of daughter cells through epigenetic mechanisms, known as epi-polarity. Changes in epi-polarity are linked to alterations in chromatin architecture and may contribute to the functional decline of aging HSC (Mejia-Ramirez et al., 2020).

Although single-cell sequencing data have covered various HSC microenvironments from formation to aging, there is still a lack of research that integrates these data and systematically compares the dynamic changes in cell communication networks. This study aims to collect HSC-related single-cell transcriptome datasets, reconstruct single-cell atlases of different hematopoietic microenvironments, and explore the dynamic patterns of cell communication therein. Understanding the molecular mechanisms of hematopoietic niche changes during aging may provide therapeutic targets to maintain HSC function and promote healthy aging, which may benefit hematology and regenerative medicine research.

## 2 Materials and methods

### 2.1 Data collection

All single cell RNA-seq data used in this study are publically available, including 2 datasets (GSE135202, GSE162950, 7 samples) for AGM (Zeng et al., 2019; Calvanese et al., 2022), 4 datasets (GSE155259, GSE162950, CRA002443, E-MTAB-7407, 31 samples) for FL (Popescu et al., 2019; Wang et al., 2020; Roy et al., 2021; Calvanese et al., 2022), 3 datasets (GSE155259, HRA002414, E-MTAB-9389, 20 samples) for FBM (Jardine et al., 2021; Roy et al., 2021; Zheng et al., 2022), GSE245108 (16 samples) for young adult bone marrow (YBM) (Zhang et al., 2024), GSE253355 (12 samples) for aged/old adult bone marrow (OBM) (Bandyopadhyay et al., 2024). The detailed information on datasets used in this study can be found in Supplementary Table S1.

### 2.2 Single-cell RNA-seq data processing

The processed raw count matrix of each dataset was downloaded from GEO, ArrayExpress or NGDC. If the count matrix is not available, raw sequencing data were downloaded and reads were mapped to the human reference genomes (refdata-gex-GRCh38-2020-A) using CellRanger (v7.1). All count matrixes were loaded into Seurat (v4.3) (Hao et al., 2021) for downstream analysis. All samples were filtered to retain high-quality cells based on the following criteria: cells with 500–8,000 detected genes, a total UMI count ≤50,000, and a mitochondrial gene expression percentage ≤10%. Samples were grouped into 5 groups: AGM (4–6 weeks), FL (4–19 weeks), FBM (10–19 weeks), YBM (21–34 years old) and OBM (52–74 years old). We integrated samples in each group and removed batch effects using Harmony (v1.2) (Korsunsky et al., 2019). Marker genes were identified using FindAllMarkers with default parameters. Cell types were annotated according to the marker genes (Supplementary Table S2) provided in related literature and the DISCO database (Li et al., 2022).

### 2.3 Single-cell trajectory analysis

To investigate the transcriptional dynamics during HSC maturation, we reconstructed the developmental trajectory using Monocle (v2.32) (Qiu et al., 2017). HLF + cells were extracted from the HSPC populations in the AGM, FL, and FBM datasets to focus on the HSC lineage. HLF is highly enriched in and specific to HSCs compared to other hematopoietic populations (Lehnertz et al., 2021; Calvanese et al., 2022). Differentially expressed genes (DEGs) along the HSC maturation trajectory were identified using the differentialGeneTest function in Monocle. Genes with a p-value or q-value ≥0.05 were filtered out to obtain a set of significant DEGs. These DEGs were then clustered and visualized using a heatmap generated by the plot_pseudotime_heatmap function.

### 2.4 Cell-cell communication analysis

CellChat (v2.1, https://github.com/jinworks/CellChat) (Jin et al., 2021; Jin et al., 2024) was employed to infer cell-cell communication via ligand-receptor interactions using annotated Seurat objects containing expression data and cell type annotations from the AGM, FL, FBM, YBM, and OBM. The analysis focused on protein-mediated interactions, with the CellChat database curated to exclude non-protein signaling pathways. Interaction number and strength was calculated for individual ligand-receptor pairs between each pair of cell types. Specifically, for each ligand-receptor pair, CellChat first calculates the communication probability by multiplying the average expression of the ligand in the sender cell type with the average expression of the receptor in the target cell type. To determine the overall interaction strength between two cell types, the interaction strengths of individual ligand-receptor pairs are aggregated. The analysis concentrated on interactions where HSPC were either sending or receiving signals. For each significant ligand-receptor pair, if the ligand is expressed by HSPCs and the receptor is expressed by another cell type, HSPCs are designated as the signal-sending cell in that interaction. Conversely, if the receptor is expressed by HSPCs and the ligand is expressed by another cell type, HSPCs are designated as the signal-receiving cell. This allows for a systematic categorization of HSPC signaling roles based on the expression patterns of ligands and receptors. Comparative analysis of CellChat results was conducted across developmental stages (AGM, FL, and FBM) and the aging continuum (FBM, YBM, and OBM). Changes in interaction strength of specific ligand-receptor pairs, cell type pairs, and signaling pathways were examined across various stages of HSC maturation and aging. The results are visualized using the “ligand-receptor dotplot” and “information flow plot” functions. The LR dotplot displays the communication probabilities of selected ligand-receptor pairs in different stages as a heatmap, allowing for easy identification of stage-specific changes in interaction strength. The information flow for a signaling pathway is calculated by summing up the communication probabilities among all pairs of cell groups in the inferred network. To obtain the relative information flow, CellChat normalizes these raw values by dividing each value by the sum of all information flow values across pathways. This normalization step ensures that the relative information flow values sum up to 1, creating a probability distribution that reflects the relative contribution of each pathway to the overall communication network.

### 2.5 Driving ligand prediction analysis

NicheNet (v2.1) (Browaeys et al., 2020) was employed to predict ligands potentially influencing the DEGs between YBM and OBM HSPC using integrated and annotated scRNA-seq data from YBM and OBM. Genes expressed in fewer than 10% of HSPC or fewer than 5% of sender cells within the HSC niche were excluded. The gene set of interest consisted of DEGs between YBM and OBM HSPC, while the background gene set included all genes expressed in OBM HSPC. Ligand activity scores, ligand expression fold change in sender cells, and the regulatory potential of targeted DEGs were used to prioritize and select the regulatory networks of interest.

### 2.6 Functional enrichment analysis

The Gene Ontology (GO) enrichment analysis was performed using clusterProfiler (v4.6) (Xu et al., 2024). The analysis was conducted separately for upregulated and downregulated DEGs. GO terms with an adjusted p-value (q-value) ≤ 0.05 were considered significantly enriched.

## 3 Results

### 3.1 The continuous single-cell landscapes of HSC niche

**FIGURE 1:**
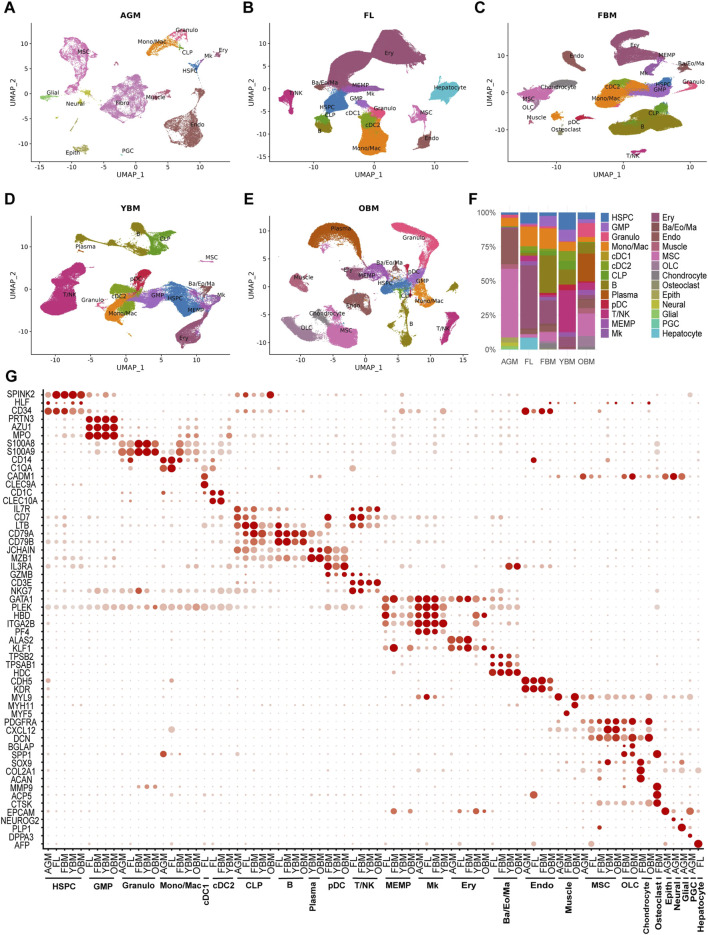
Integration and annotation of the single-cell atlases of HSC niches. **(A–E)** UMAP visualizations of single-cell atlases including AGM, FL, FBM, YBM and OBM. **(F)** The proportions of 26 distinct cell types at different stages. **(G)** Marker expression of cell types in different stages. HSPC, hematopoietic stem/progenitor cells; GMP, granulocyte-monocyte progenitors; Granulo, granulocytes; Mono/Mac, monocytes/macrophages; cDC1, conventional dendritic cells type 1; cDC2, conventional dendritic cells type 2; CLP, common lymphoid progenitors; pDC, plasmacytoid dendritic cells; MEMP, megakaryocyte-erythroid-mast cell progenitors; Mk, megakaryocytes; Ery, erythrocytes; Ba/Eo/Ma, basophils/eosinophils/mast cells; Endo, endothelial cells; MSC, mesenchymal stem/stromal cells; Fibro, fibroblasts; OLC, osteolineage cells; Epith, epithelial cells; PGC, primordial germ cells.

To comprehensively characterize the molecular and cellular changes within the hematopoietic niche during development and aging, we analyzed 11 single-cell RNA sequencing (scRNA-seq) datasets encompassing the main hematopoietic microenvironments. These datasets included samples from the AGM region, FL, FBM, YBM, and OBM. After stringent quality control measures, batch effect correction, and data integration, we constructed five single-cell atlases of the HSC niche, comprising 23,732 cells from AGM, 217,693 cells from FL, 134,261 cells from FBM, 97,614 cells from YBM, and 82,742 cells from OBM (Figures 1A–E). Using markers from literature and databases, we annotated 26 major cell types in these cell atlases, including HSPC, other hematopoietic cells, endothelial cells, MSC, epithelial cells, muscle cells, neural cells, etc. Notably, the proportions of these cell types exhibited marked variations across the five stages (Figure 1F). These changes in niche cell composition likely play a crucial role in regulating HSC function and fate at distinct developmental stages. For example, the exclusive presence of primordial germ cells (PGC) in the AGM dataset suggests that these cells may contribute to the unique properties of the AGM niche in supporting HSC emergence and expansion. PGCs have been reported to secrete factors such as BMP4 and WNT3A, which are known to promote HSC development and self-renewal (Zuo et al., 2021; Esfahani et al., 2024). Similarly, the unique presence of hepatocytes in the FL dataset indicates their specific role in supporting HSC expansion and differentiation during fetal hematopoiesis. Hepatocytes have been shown to produce cytokines like SCF and TPO, which are essential for HSC proliferation and survival (Lee et al., 2021; Lee et al., 2022). To validate the robustness of our cell type annotations, we examined the expression patterns of canonical markers for each identified cell type across the five groups. Remarkably, the expression profiles of these markers remained highly consistent in the corresponding cell types across all developmental and aging stages (Figure 1G).

### 3.2 The evolving niche interactome from HSC ontogeny, expansion to maturation

We reconstructed the developmental trajectory of HSC in the AGM, FL, and FBM regions and compared the communication patterns among these three regions to comprehensively understand the evolving niche interactome and its adaptation to the needs of maturing HSC (Supplementary Figures S1A–C). DEGs that show regular changes along the HSC maturation trajectory can be categorized into upregulated and downregulated genes (Supplementary Figure S1D; Supplementary Table S3). Upregulated genes, including CD48, CD52, CD74, CD83, and transcription factors like JUN, EGR1, and GATA2, play significant roles in facilitating HSC maturation. CD molecules such as CD48 and CD74 are involved in cell adhesion and signaling, crucial for HSC niche interactions and promoting maturation (Boles et al., 2011; Becker-Herman et al., 2021). Upregulated genes are primarily associated with immune response regulation and hematopoietic activation Supplementary Figure S1E; Supplementary Table S4).

**FIGURE 2:**
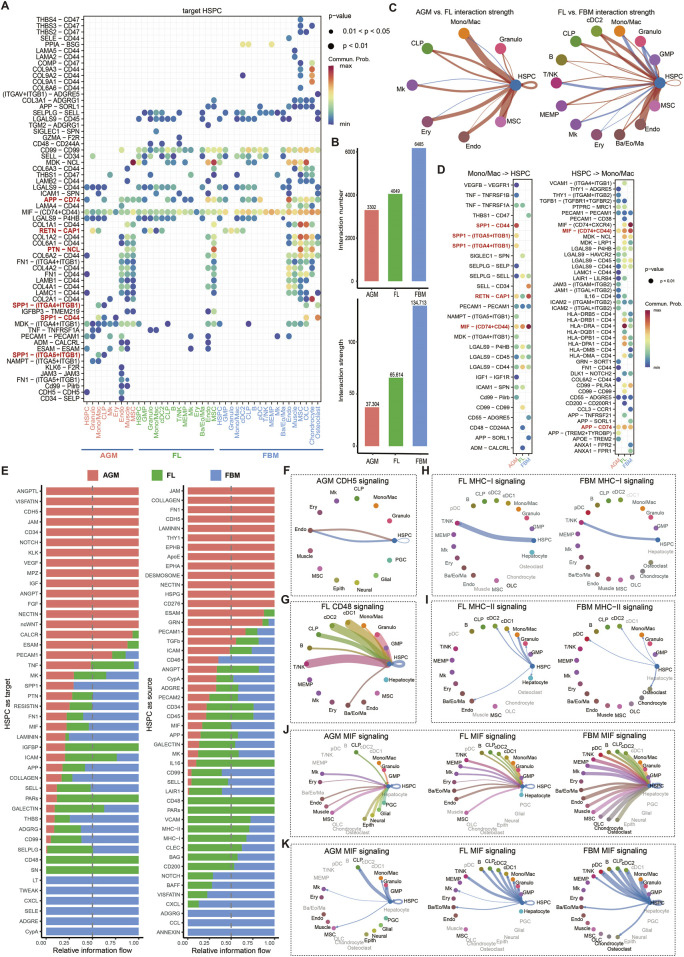
Dynamic cell-cell communication patterns during HSC maturation. **(A)** Ligand-receptor communication probabilities from niche cells to HSPC in AGM, FL and FBM. **(B)** Quantification of the overall interaction number and strength in the AGM, FL, and FBM niches. **(C)** Comparative analysis of interaction strength between AGM and FL, and between FL and FBM. Red lines indicate an increase in interaction strength in the FL compared to the AGM, or in the FBM compared to the FL. Blue lines indicate a decrease in interaction strength across the same developmental transitions. **(D)** Detailed representation of ligand-receptor pairs between HSPC and Mono/Mac. **(E)** The relative information flow of each signaling pathway received or sent by HSPC in the AGM, FL, and FBM. **(F–K)** Circle plots illustrating selected signaling pathways received or sent by HSPC, highlighting the dynamic nature of cell-cell communication during HSC maturation.

To investigate the dynamic niche interactions during HSC ontogeny and maturation, we employed CellChat (Jin et al., 2021) to infer cell-cell communication via ligand-receptor interactions. Ligand-receptor analysis targeting HSPC revealed dynamic cell communication regulation during development and maturation (Figure 2A). We identified numerous specific ligand-receptor pairs in the AGM and FBM microenvironments, with FL serving as an intermediate stage, exhibiting similar ligand-receptor relationships to both AGM and FBM. Aggregating the number and strength of interactions between cells in HSC niches revealed an increase in these interactions during HSC maturation (Figure 2B). Examining the interactions of each cell type targeting HSPC separately, we observed that the interaction strength of all cell types targeting HSPC increased from AGM to FL, while most cell types exhibited increased interaction strength from FL to FBM (Figure 2C).

Mono/Mac-derived SPP1 binding to CD44 on HSPC exhibits the highest interaction strength in AGM but is not detected in FL and FBM (Figure 2D). This binding activates PI3K/Akt and MAPK signaling pathways, promoting HSC proliferation and survival during early hematopoietic development (Herishanu et al., 2011; Li et al., 2023). Additionally, Mono/Mac-derived SPP1 binds to ITGA5-ITGB1 and ITGA4-ITGB1 co-receptors in AGM but not in FL and FBM. Activation of FAK and Src family kinases downstream of ITGA5-ITGB1 supports HSC adhesion to the extracellular matrix and differentiation, while NF-kB and MAPK signaling pathways activated by ITGA4-ITGB1 contribute to HSC homing and maintenance (Khurana et al., 2016; Krenn et al., 2022). These interactions highlight the diverse regulatory roles of macrophage-derived SPP1 and its co-receptor complexes in governing HSC emergence rather than expansion and maturation in FL and FBM. Mono/Mac-secreted resistin (RETN) binding to CAP1 (calcyphosin) receptor on HSPC significantly increases in interaction strength in FBM compared to FL. RETN-CAP1 binding activates the cAMP/PKA pathway in various cell types, including immune cells and adipocytes (Lee et al., 2014). Additionally, the interaction between amyloid precursor protein (APP) on endothelial cells and CD74 on HSPC progressively strengthens from AGM to FL and FBM (Figure 2A). CD74 can regulate the expression of transcription factors including KLF4, IRF8, and CEBPA, which are known to regulate HSC maintenance (Becker-Herman et al., 2021). The interaction between pleiotrophin (PTN) on MSC and nucleolin (NCL) on HSPC shows a significant increase in interaction strength from the AGM to the FL, with the interaction stabilizing from the FL to the FBM. PTN-NCL binding primarily activates the Wnt and BMP signaling pathway, which is crucial for guiding tissue renewal and regeneration (Reister et al., 2019; Reister et al., 2021).

We also compared the relative information flow of each cell-cell communication signaling among AGM, FL and FBM. In the AGM niche, several pathways are notably specific and crucial for HSC emergence, including ANGPTL, VISFATIN, CDH5, JAM, CD34, NOTCH, KLK, VEGF, MPZ, IGF, ANGPT, FGF, NECTIN, and ncWNT (Figures 2E, F). Many of these pathways, such as NOTCH signaling, are derived from endothelial cells and play a vital role in the EHT process and HSC fate determination (Lomeli and Castillo-Castellanos, 2020; Cai et al., 2024). These pathways ensure a well-structured niche that supports the initial emergence and development of HSC, preparing them for further migration and adhesion in the FL. In the FL, CD48 signaling is highly specific, with CD48 serving as a co-stimulatory molecule targeting CD244A on HSPC. CD48 is primarily expressed in HSPC, granulocyte, cDC1, cDC2, CLP, and T/NK cells (Figure 2G). The CD48-CD244A interaction activates cytokine signaling such as IFNγ, supporting HSC proliferation and function (Boles et al., 2011). The signaling pathways specific to the FBM that target HSPC including LT, TWEAK, CXCL, SELE, ADGRE, and CypA. CypA (cyclophilin A or PPIA) is involved in protein folding and stabilization, influencing HSC survival and differentiation through modulation of cellular stress responses. Depletion of PPIA can accelerate HSC aging (Maneix et al., 2024).

The expression of MHC-I and MHC-II molecules on HSPC is a hallmark of their initial maturation (Supplementary Figures S1E, F). In FL and FBM, MHC-I signaling enables HSPC to target T/NK cells, with the interaction strength decreasing over time (Figure 2H). This decline may reflect a reduced role for mature HSC in directly engaging T/NK cells, potentially indicating a shift in their functional requirements during maturation (Calvanese et al., 2022). MHC-II signaling in FL and FBM involves interactions with various cells, including DCs and other antigen-presenting cells, with a general decrease in interaction strength (Figure 2I). This broad interaction profile highlights the role of MHC-II in shaping the immune environment around HSC, potentially facilitating their integration into the immune system. Notably, the MIF signaling pathway exhibits a progressively increasing and robust interaction across the AGM, FL, and FBM regions during HSC maturation (Figures 2A, J, K). MIF (macrophage migration inhibitory factor) secreted by all cell types targets HSPC mainly through the CD74-CD44 co-receptor complex (Becker-Herman et al., 2021), demonstrating the growing importance of this signaling axis as HSC transition through their developmental stages.

### 3.3 The alteration of niche interactome during HSC aging

**FIGURE 3:**
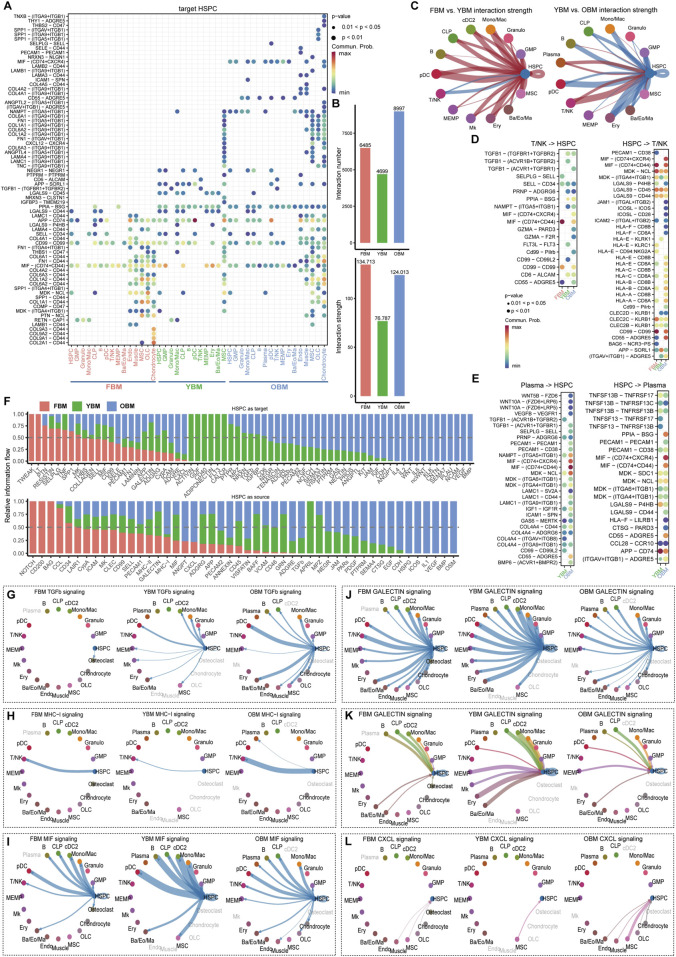
Dynamic cell-cell communication patterns during HSC aging. **(A)** Ligand-receptor communication probabilities from niche cells to HSPC in FBM, YBM and OBM. **(B)** Quantification of the overall interaction number and strength in the FBM, YBM and OBM niches. **(C)** Comparative analysis of interaction strength between FBM and YBM, and between YBM and OBM. **(D)** Detailed representation of ligand-receptor pairs between HSPC and T/NK. **(E)** Detailed representation of ligand-receptor pairs between HSPC and plasma cells. **(F)** The relative information flow of each signaling pathway received or sent by HSPC in the FBM, YBM and OBM. **(G–L)** Circle plots illustrating selected signaling pathways received or sent by HSPC, highlighting the dynamic nature of cell-cell communication during HSC aging.

To investigate the alteration of cell-cell communication in the HSC niche during aging, the interaction was evaluated in FBM, YBM, and OBM, and then compared among the three regions to identify changes in communication patterns between OBM and YBM, with the FBM serving as a reference for the relatively stable development of HSC during fetal stage and young adulthood. The overall interaction strength is decreased from FBM to YBM, which may suggest a decline in supportive interactions necessary for maintaining HSC function (Figures 3A, B). The altered cell-cell communication patterns observed in the aging HSC niche have significant implications for HSC function and biological behavior. As HSC age from YBM to OBM, the increased interaction strength may reflect an adaptive response aimed at preserving HSC functionality (Figures 3C, D). The consistent increase in interaction strength from plasma cells and T/NK cells targeting HSPC highlights the critical role of immune cells in modulating the aging HSC niche. The interaction of T/NK cells with HSPC through the GZMA-PARD3 and GZMA-F2R ligand-receptor pairs, specific to OBM and absent in YBM (Figure 3D), induces nitric oxide production, enhancing CXCL12-CXCR4–induced motility and rapid stem and progenitor cell mobilization (Gur-Cohen et al., 2015). The presence of plasma-HSPC interactions such as WNT10A-(FZD6+LRP6), WNT10A-(FZD6+LRP5), and WNT5B-FZD6 in OBM, but not in YBM (Figure 3E), indicates a shift towards more complex signaling in older HSC niches. These Wnt signaling pathways have been implicated in regulating HSC self-renewal, differentiation, and aging. For example, increased Wnt signaling has been shown to induce HSC aging by promoting symmetric division and reducing HSC quiescence (Abidin et al., 2015; Liu et al., 2019). Comparing the ligand-receptor pathways of FBM, YBM, and OBM revealed an enrichment of interleukin signals (IL1, IL2, and IL4) in OBM (Figure 3F), likely contributing to HSC aging by promoting an inflammatory and less regenerative niche (He and Wang, 2021; Caiado and Manz, 2024). The increased exposure of HSCs to inflammatory cytokines in the aging niche may therefore contribute to their functional decline and reduced ability to maintain blood homeostasis.

The increased targeting of various cell types by HSC through the TGF-β pathway from YBM to OBM, absent in FBM, suggests a significant alteration in the regulatory environment associated with HSC aging (Figure 3G). TGF-β signaling has been shown to play a critical role in maintaining HSC quiescence and preventing excessive proliferation (Chambers et al., 2007). The altered TGF-β signaling in the aging niche may disrupt this balance, leading to increased HSC cycling and eventual exhaustion. The enhancement of HSC targeting to T/NK cells via the MHC-I pathway from YBM to OBM (Figure 3H) may reflect increased immune surveillance and clearance activity (Weiskopf et al., 2016). Additionally, the chronic exposure of HSCs to immune stress may lead to telomere shortening and other age-related cellular changes, further contributing to their functional decline. The weakening of MIF pathway interactions (Figures 3A, I) and the reduction in interactions between HSPC and other cell types via the galectin pathway from YBM to OBM (Figures 3J, K) suggest a broader decline in the efficacy of these signaling pathways within the aging HSC niche. The increase in CXCL12-CXCR4 interactions between MSC and HSPC from YBM to OBM (Figure 3L) indicates an enhanced retention and localization of HSC within the niche. While this increased retention may initially serve to protect HSCs against oxidative stress accumulated during aging (Zhang et al., 2016), it may also lead to altered stem cell behavior and function over time. The prolonged exposure of HSCs to the aging niche environment, characterized by chronic inflammation and altered signaling, may ultimately contribute to their functional decline and impaired regenerative capacity.

### 3.4 The driving ligands and target regulators during HSC aging

**FIGURE 4:**
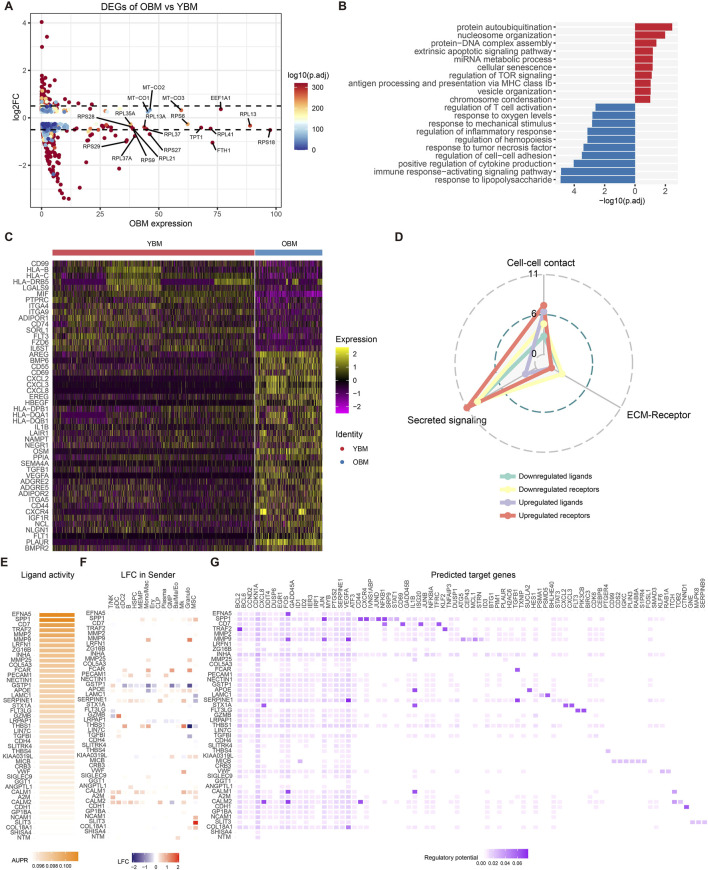
Alterations in gene expression and driving ligands during HSC aging. **(A)** Volcano plot showing DEGs between HSPC from OBM and YBM. **(B)** GO enrichment analysis results for upregulated (blue) and downregulated (red) genes. Downregulated GO terms are colored in red. **(C)** Heatmap displaying the expression patterns of ligands and receptors within the DEGs. **(D)** Distribution of signaling types for ligand-receptor pairs identified in the DEGs. **(E)** Ligand activities for the top 30 ligands predicted by NicheNet. **(F)** The log2FoldChange of top 30 ligands in common cell types of YBM and OBM. **(G)** Predicted target genes for the top 30 ligands, with upregulated target genes in OBM colored in blue and downregulated genes colored in red.

To characterize the changes in HSC at the transcriptomic level during aging and the alterations in the niche interactome of old HSC compared to young HSC, DEGs of HSPC between OBM and YBM were identified (Figure 4A; Supplementary Table S5). Upregulated genes involved in ribosomal function (RPS18, RPS27, RPL27A) and iron metabolism (FTH1, FTL) indicate increased ribosomal activity and attempts to manage oxidative stress, respectively (Yi et al., 2024). Enrichment analysis of upregulated and downregulated genes revealed increased inflammatory and stress responses associated with aging and cellular senescence (Figure 4B; Supplementary Table S6), such as response to lipopolysaccharide and positive regulation of cytokine production, suggesting heightened immune activation and inflammation (Kim et al., 2016). The over-representation of the “myeloid cell differentiation” term in the OBM suggests that there is an increased activity or propensity towards myeloid lineage commitment in the hematopoietic system during aging (Supplementary Table S6). This finding is consistent with the well-documented phenomenon of age-related myeloid skewing, where the balance of hematopoietic output shifts towards the myeloid lineage at the expense of lymphoid cell production (Oduro et al., 2012; Dorshkind et al., 2020). Analysis of upregulated and downregulated ligands/receptors showed a consistent trend with CellChat analysis (Figure 4C), with most altered interactions during HSC aging belonging to secreted signaling (Figure 4D).

NicheNet was used to infer the activity of ligands targeting the DEGs in HSPC and identify ligands driving HSC aging (Figure 4E). SPP1 was found to be significantly upregulated in MSC, leading to increased expression of its target genes JUN and JUND in HSPC (Figures 4F, G), consistent with the intensification of SPP1 signaling activity targeting HSPC from YBM to OBM during HSC aging (Figure 3A). The increased expression of JUN and JUND may contribute to HSC dysfunction by exacerbating inflammation and skewing myeloid differentiation (Matteini et al., 2021). MMP9, most significantly upregulated in Mono/Mac and granulocytes (Figure 4F), affects HSC by increasing the expression of FOS, JUN, and VEGFA (Figure 4G), facilitating inflammatory signaling and altering the HSC niche, contributing to age-related functional decline (Saw et al., 2019). Increased FCAR expression in Mono/Mac, GMP, and granulocytes leads to elevated TGFB1 levels in HSPC, which may impair HSC function and promote aging-related changes (Blank and Karlsson, 2015). These observations indicate that changes in ligand expression from niche cells significantly impact HSC function, promoting the aging process through mechanisms involving inflammation, oxidative stress, and cellular remodeling. Inhibiting these niche-derived ligands targeting HSPC could offer a promising strategy to mitigate HSC aging and preserve their function.

## 4 Discussion

In this study, we comprehensively investigated the changes in gene expression and signaling pathways induced by cell-cell communication in HSC during maturation and aging. By collectively analyzing genes and ligand-receptor pathways with similar trends during these processes, we aimed to infer their potential roles in promoting HSC maturation and aging.

Our findings reveal that several genes and pathways exhibit significant changes during maturation and aging, reflecting their critical roles in these processes. Genes such as CD99, CD74, PTPRC, LGALS9, FTH1, RPS27, JUND, SRGN, and CHMP1B are upregulated during maturation but downregulated during aging, indicating a potential decline in immune response, iron metabolism, ribosomal function, and stress responses in aged HSC (de Haan and Lazare, 2018; Hammond et al., 2023). The MIF-(CD74^+^CD44) signaling pathway, crucial for HSC proliferation and survival (Becker-Herman et al., 2021), is upregulated during maturation but downregulated during aging, highlighting a loss of these protective mechanisms in aged HSC. In contrast, genes such as TGFB1, NCL, GAPDH, and ACTB are downregulated during maturation but upregulated during aging, suggesting that their increased expression during aging may contribute to the functional decline of HSC by promoting stress responses and metabolic dysregulation (Mahotka et al., 2018; Shiroshita et al., 2023). Interestingly, our analysis revealed significant changes in the expression of GAPDH and ACTB, two genes commonly used as housekeeping genes in RT-qPCR experiments. The observed downregulation of these genes during maturation and their upregulation during aging highlight the importance of carefully selecting reference genes when studying HSC biology across different developmental and age-related stages.

Furthermore, genes and pathways consistently upregulated during both maturation and aging, such as B2M, CXCL8, PTN, FN, LAMININ, COLLAGEN, and THBS signaling pathways, suggest their fundamental roles in maintaining HSC function through cell adhesion and interaction with the extracellular matrix (Lee-Thedieck et al., 2022). These observations collectively indicate that changes in gene expression and signaling pathways from niche cells significantly impact HSC function, promoting the aging process through mechanisms involving inflammation, oxidative stress, and cellular remodeling. Notably, targeting these niche-derived ligands could potentially inhibit HSC aging, offering new avenues for therapeutic intervention in age-related hematopoietic decline (Zhang et al., 2020). Our results also suggest a potential link between the age-dependent changes in HSC-niche communication and the altered HSC mobilization potential observed during aging. Previous studies have shown that aged HSCs exhibit reduced mobilization efficiency in response to stimuli such as G-CSF (Xing et al., 2006; Geiger et al., 2007). The altered signaling pathways and communication dynamics between HSCs and their niche components, as revealed in our study, could contribute to this age-related decline in mobilization potential. For example, the increased TGF-β signaling in the aged niche may enhance HSC adhesion and retention, making them less responsive to mobilizing agents (Blank et al., 2008).

It is important to acknowledge the limitations of our study. First, the data used in this study were pooled from various sources that employed different pre-selection methods, primarily based on flow cytometry. These differences in cell sorting strategies, marker profiles, and gating parameters may introduce biases in the interpretation of cell type composition, and hinder the accurate comparison of population sizes and cell-cell communication properties across different developmental stages and time points. Consequently, the direct comparison of cell communication flow among datasets obtained using distinct cell selection methods may not fully reflect the true *in vivo* cell communication dynamics within the HSC niche. Furthermore, the cell-cell communication probability analysis relies on RNA expression data, which may not comprehensively represent the intricate nature of ligand-receptor interactions at the protein level. The presence of mRNA for a specific ligand-receptor pair does not guarantee their functional interaction, as post-transcriptional modifications, protein localization, and other regulatory mechanisms can influence the actual protein-level communication between cells. Additionally, the mere expression of a ligand by one cell and its corresponding receptor by another does not necessarily indicate their proximity within the niche. The spatial arrangement and niche context of cells *in vivo* may differ from the assumptions based on mRNA expression patterns, and cells expressing complementary ligand-receptor pairs may not physically interact in the niche. Besides, the pooling of results from multiple samples in our study precludes the inclusion of statistical standard deviation ranges in the figures and graphs. This limitation hinders the assessment of inter-sample variation and the dispersion of data points around the mean, which could provide valuable information about the robustness and reproducibility of the observed trends.

In conclusion, our study provides a comprehensive understanding of the molecular mechanisms underlying HSC maturation and aging, highlighting the crucial role of cell-cell communication in regulating HSC function. Despite the limitations mentioned above, these findings lay the foundation for developing targeted therapies to mitigate age-related hematopoietic decline and maintain healthy hematopoiesis throughout life. Future studies using advanced single-cell techniques and *in vivo* validation of the identified ligand-receptor interactions will further refine our understanding of the complex interplay between HSCs and their niche components during maturation and aging.

## Data Availability

The original contributions presented in the study are included in the article/Supplementary Material, further inquiries can be directed to the corresponding author.

## Supplementary material

SUPPLEMENTARY FIGURE S1 Intrinsic ligand and receptor expression patterns on HSC maturation trajectory. **(A)** HLF + HSC trajectory constructed using Monocle2, revealing the developmental progression of HSC. HLF is a marker gene that has been shown to be able to track HSC across microenvironments. **(B)** Pseudotime inference of the HLF + HSC trajectory. **(C)** Split view of the HLF + HSC trajectory ordered by different developmental stages. **(D)** Heatmap showing two clusters of DEGs along the HSC trajectory. **(E)** GO enrichment analysis results for the two clusters of DEGs. Red bars indicate the upregulated cluster. Blue bars indicate the downregulated cluster. **(F)** Expression trends of selected ligands or receptors from the DEGs.
