# Supplementary figures and images for: Deciphering the evolving niche interactome of human hematopoietic stem cells from ontogeny to aging
Source: Front Mol Biosci. 2024 Dec 4;11:1479605. doi: 10.3389/fmolb.2024.1479605 (PMC11652281; doi:10.3389/fmolb.2024.1479605)

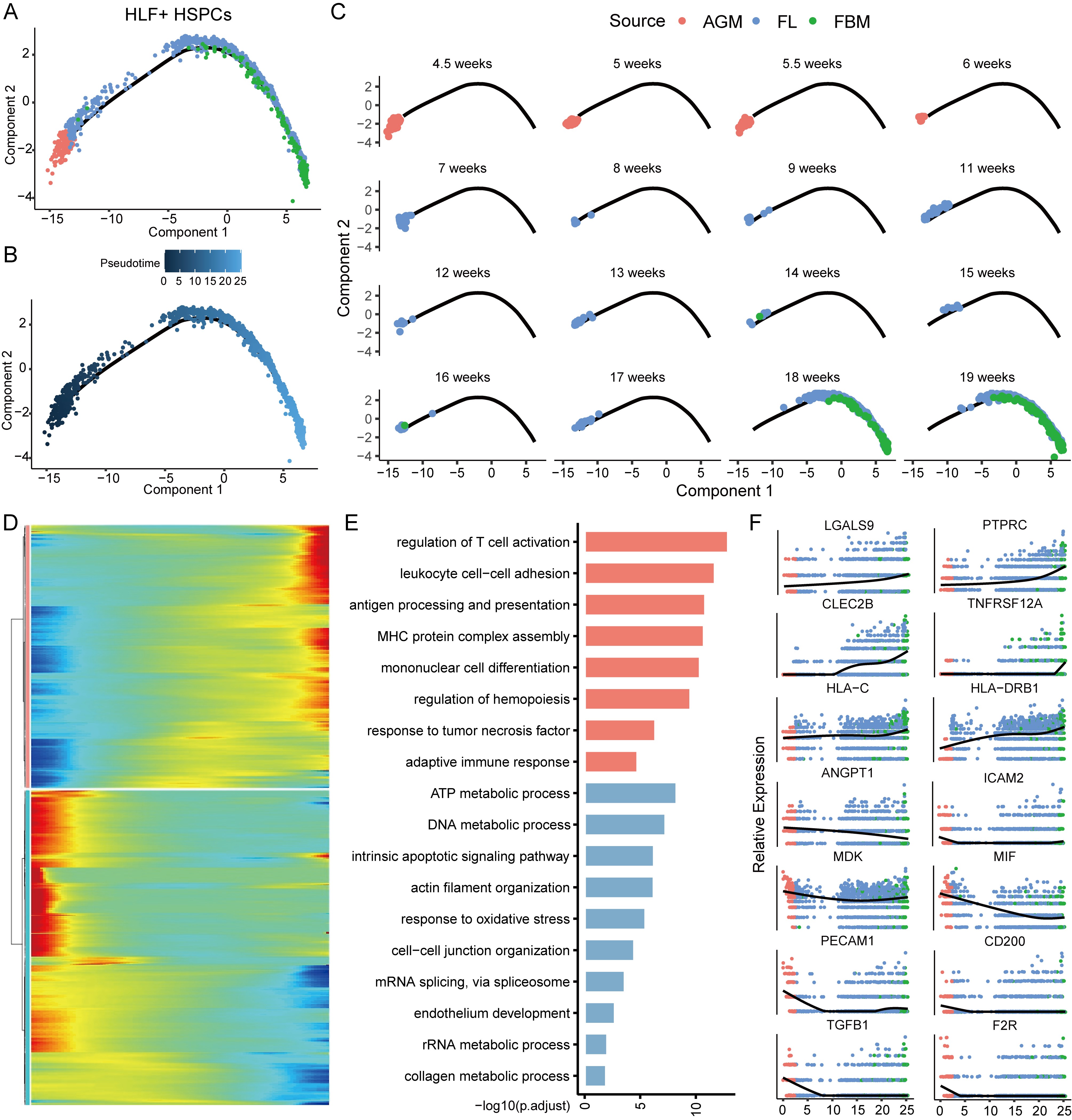

Supplement: Supplementary file 3 [file Image1.jpeg]
